# Anti-Inflammatory Effects of the Nicotinergic Peptides SLURP-1 and SLURP-2 on Human Intestinal Epithelial Cells and Immunocytes

**DOI:** 10.1155/2014/609086

**Published:** 2014-05-04

**Authors:** Alex I. Chernyavsky, Valentin Galitovskiy, Igor B. Shchepotin, Sergei A. Grando

**Affiliations:** ^1^Department of Dermatology, University of California, 134 Sprague Hall, Irvine, CA 92697, USA; ^2^National Cancer Institute, Kiev 03022, Ukraine; ^3^Department of Biological Chemistry, University of California, 134 Sprague Hall, Irvine, CA 92697, USA; ^4^Institute for Immunology, University of California, 134 Sprague Hall, Irvine, CA 92697, USA

## Abstract

A search for novel and more efficient therapeutic modalities of inflammatory bowel disease (IBD) is one of the most important tasks of contemporary medicine. The anti-inflammatory action of nicotine in IBD might be therapeutic, but its toxicity due to off-target and nonreceptor effects limited its use and prompted a search for nontoxic nicotinergic drugs. We tested the hypothesis that SLURP-1 and -2—the physiological nicotinergic substances produced by the human intestinal epithelial cells (IEC) and immunocytes—can mimic the anti-inflammatory effects of nicotine. We used human CCL-241 enterocytes, CCL-248 colonocytes, CCRF-CEM T-cells, and U937 macrophages. SLURP-1 diminished the TLR9-dependent secretion of IL-8 by CCL-241, and IFN**γ**-induced upregulation of ICAM-1 in both IEC types. rSLURP-2 inhibited IL-1**β**-induced secretion of IL-6 and TLR4- and TLR9-dependent induction of CXCL10 and IL-8, respectively, in CCL-241. rSLURP-1 decreased production of TNF**α** by T-cells, downregulated IL-1**β** and IL-6 secretion by macrophages, and moderately upregulated IL-10 production by both types of immunocytes. SLURP-2 downregulated TNF**α** and IFN**γ**R in T-cells and reduced IL-6 production by macrophages. Combining both SLURPs amplified their anti-inflammatory effects. Learning the pharmacology of SLURP-1 and -2 actions on enterocytes, colonocytes, T cells, and macrophages may help develop novel effective treatments of IBD.

## 1. Introduction

A search for novel and more efficient therapeutic modalities of inflammatory bowel disease (IBD) is one of the most important tasks of contemporary clinical and experimental medicine. Both ulcerative colitis (UC) and Crohn's disease (CD) are epidemiologically related to smoking [1–4]. Most patients with UC are nonsmokers, and patients with a history of smoking usually acquire their disease after they have stopped smoking [5–7]. Upon cessation of smoking, patients with UC experience more severe disease progression that can be ameliorated by returning to smoking [8–10]. In contrast, patients with CD experience severe disease when smoking, requiring an immediate and complete cessation of any tobacco usage [3, 11]. Nicotine administration in transdermal patches or enema inhibits inflammation associated with UC [8, 12–16]. Nicotine also exhibits a local therapeutic effect in CD [17], despite the fact that smoking worsens this disease. It is believed that the therapeutic effects of nicotine in IBD are mediated by the nicotinic acetylcholine (ACh) receptors (nAChRs) of gut immune cells that inhibit production of inflammatory mediators and correct specific alterations in cell cycle responses [18–20]. We have previously demonstrated that nicotinic agonists abrogate PHA-dependent upregulation of TNF*α* and IFN*γ* receptors (IFN*γ*R) in the human leukemic T-cell line CCRF-CEM (CEM) [21] and downregulate lipopolysaccharide- (LPS-) induced production of the proinflammatory cytokines IL-6 and IL-18 but upregulated IL-10 in human macrophage-like U937 cells [22]. On the other hand, recent research has conclusively demonstrated that dysregulation of intestinal epithelial cells (IEC) plays an important role in the pathogenesis of IBD [23], but the therapeutic modalities that can effectively correct function of these cells remain unknown. An important role of IEC response to nicotinic drugs in IBD has been suggested by the presence of fully developed, functional ACh axis in the intestinal epithelium, with its nicotinic arm controlling intestinal absorption, permeability, mucociliary activity, and mucin secretion, as well as IEC viability, proliferation, migration, and cohesion [24–38]. Therefore, modulation of the nicotinergic anti-inflammatory pathway is considered as a novel therapeutic target for IBD [12, 39–41]. Clinical trials of nicotine formulations, however, revealed severe side effects from therapeutic doses of nicotine [12, 42], which prompted a search for nontoxic nicotinergic agents that can mimic anti-inflammatory effects of nicotine in patients with IBD.

A novel paradigm of cell regulation via nAChRs has been discovered in studies of the autosomal recessive disease palmoplantar keratoderma featuring mutation of secreted mammalian Ly-6/urokinase plasminogen activator receptor-related protein- (SLURP-) 1 and impaired T-cell activity [43]. SLURP-2 expression was also discovered in the skin [44]. While various subtypes of nAChRs can be involved in the physiological regulation of cell functions by SLURPs, the biological effects of SLURP-1 are predominantly mediated by *α*7 nAChR and those of SLURP-2 by non-*α*7 nAChRs [45]. Cell function and gene expression studies [46, 47] suggested that SLURPs may play important roles in regulating both epithelial cells and immunocytes. Since nicotine has been shown to alter expression of SLURP-1 in IEC [48], we hypothesized that auto/paracrine action of SLURPs on IEC may, in part, mediate the anti-inflammatory activities of nicotine in IBD.

In this study, we analyzed the roles of SLURP-1 and -2 in the physiological regulation of the key elements of the pathobiology of IBD controlling intestinal inflammation and facilitating healing of intestinal ulcers. The results demonstrated that SLURPs can abolish expression of the IBD-related mediators of inflammation in both IEC and immunocytes. Learning the pharmacology of the SLURP-1 and -2 actions on enterocytes, colonocytes, T-cells, and macrophages may therefore help develop novel effective treatments of UC and CD.

## 2. Materials and Methods

### 2.1. Cells and Reagents

Human IEC: the small intestine enterocyte cell line CCL-241 and the colonocyte cell line CCL-248, human lymphoblastoid T-cell line CEM, and human monoblastoid tumor cell line U937 were purchased from ATCC (Manassas, VA) and grown in the respective ATCC complete growth media at 37°C in a humid, 5% CO_2_ incubator. To differentiate into macrophages, the U937 cells were treated with 200 nM PMA (Sigma-Aldrich Corporation, St. Louis, MO) and allowed to adhere to tissue culture plate for 3 days [49]. The full length recombinant (r)SLURP-1 and rSLURP-2 were manufactured at Virusys Corporation (Sykesville, MD), as detailed elsewhere [50]. The previously characterized anti-SLURP-1 and -2 monoclonal antibodies 336H12-1A3 and 341F10-1F12, respectively [46, 47], were from Research and Diagnostic Antibodies (North Las Vegas, NV). Normal mouse IgG (NIgG) was obtained from Santa Cruz Biotechnology, Inc. (Santa Cruz, CA). Primary mouse antibodies to human ICAM, IL-1*β*, IL-6, IL-10, TNF*α*, and IFN*γ* receptor (IFN*γ*R) and ELISA kits for measuring human IL-6 and CXCL10 were purchased from R&D Systems (Minneapolis, MN). The IL-8 ELISA kit was from BD Biosciences (San Jose, CA). Both recombinant IL-1*β* and INF*γ* were from R&D Systems and both* E. coli* DNA and LPS from* E. coli* K12 strain (LPS-EK) were purchased from InvivoGen (San Diego, CA).

### 2.2. Quantitative Immunocytochemical Assay (QIA)

The QIA (a.k.a. in-cell western), a high throughput quantitative assay of cellular proteins, was performed* in situ*, as described in detail elsewhere [46], using the reagents and equipment from LI-COR Biotechnology (Lincoln, NE). The CCL-241, CCL-248, CEM, or U937 cells, 1 × 10^6^/well of a 96-well plate, were incubated in respective growth media with or without rSLURPs for 16 h, fixed* in situ*, washed, permeabilized with Triton solution, incubated with the LI-COR Odyssey Blocking Buffer for 1.5 h, and then treated overnight at 4°C with a primary antibody. The cells were then washed and stained for 1 h at room temperature with a secondary antibody, and expression of the protein of interest was quantitated using the LI-COR Odyssey Imaging System. Sapphire700 (1 : 1000) was used to normalize for cell number/well.

### 2.3. Statistical Analysis

Results were expressed as mean ± SD, and statistical significance was determined by ANOVA with Dunnett's posttest using the GraphPad Prism software (GraphPad Prism Software Inc., San Diego, CA). The differences were deemed significant when the calculated *P* value was <0.05.

## 3. Results

### 3.1. Anti-Inflammatory Effects of rSLURP-1 and -2 on IEC

**Figure 1:**
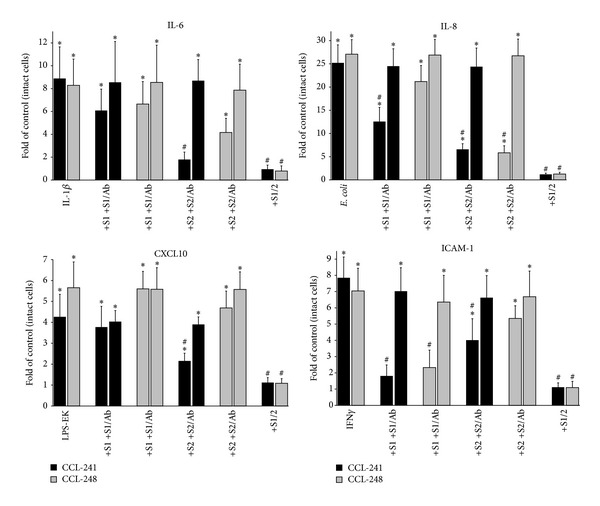
Anti-inflammatory effects of rSLURP-1 and -2 on IEC. The anti-inflammatory effects of 0.01 *μ*g/mL of rSLURP-1 (S1) and -2 (S2) on secretion of IL-6, IL-8, and CXCL10 (ELISA) and expression of ICAM-1 (QIA) by CCL-241 and CCL-248 stimulated for 16 h in a humid, 5% CO_2_ incubator at a cell density of 1 × 10^6^ cells/well with 100 U/mL of IL-1*β* (IL-6 assay), 25 *μ*g/mL of the TLR9 ligand* E. coli* DNA (IL-8), 100 ng/mL of the TLR4 ligand LPS-EK (CXCL10), or 100 U/mL of INF*γ* (ICAM-1) were measured as described in Materials and Methods. Some cells were exposed to S1 or S2 in the presence of 1 *μ*g/mL of anti-SLURP-1 or -2 monoclonal antibodies (Ab). Each experiment was performed in triplicate. Asterisk = *P* < 0.05, compared to untreated cells. Pound sign = *P* < 0.05, compared to an inflammatory stimulant given alone.

In* in vitro* experiments utilizing cultured human enterocytes and colonocytes, CCL-241 and CCL-248, respectively, we recreated an aspect of IBD pathophysiology involving the proinflammatory action of IL-1*β*, IFN*γ*, and Toll-like receptor 4- (TLR4-) and TLR9-ligands (i.e., LPS-EK and* E. coli* DNA, resp.) on intestinal epithelium [51–53]. TLR4 and TLR9 regulate cytokine secretion, cell survival, and intestinal barrier function, and their expression on IEC is upregulated in IBD [52–57]. We hypothesized that, in response to these mediators, CCL-241 and CCL-248 cells would express proinflammatory molecules eliciting mucosal homing of T-cells and recruiting other types of inflammatory cells. Exposed IEC indeed showed upregulated expression of IL-6, IL-8, CXCL10, and ICAM-1 (Figure 1).

Next, we sought to determine if rSLURP-1 or -2 can inhibit production of these proinflammatory molecules. rSLURP-1 significantly (*P* < 0.05) diminished the TLR9-dependent secretion of IL-8 by CCL-241, but not CCL-248, and the IFN*γ*-induced upregulation of ICAM-1 in both types of IEC (Figure 1). rSLURP-2 inhibited the IL-1*β*-induced secretion of IL-6 and TLR4- and TLR9-dependent induction of CXCL10 and IL-8, respectively, in CCL-241. The specificity of these effects was demonstrated by ability of anti-SLURP antibodies to abolish the inhibitory activity of corresponding rSLURP. A mixture of both nicotinergic peptides almost completely inhibited upregulated expression of all tested inflammatory molecules in both types of IEC (Figure 1), which is in keeping with the synergistic mechanisms of their biological action [58, 59].

### 3.2. Anti-Inflammatory Effects of rSLURP-1 and -2 on Immunocytes

**Figure 2:**
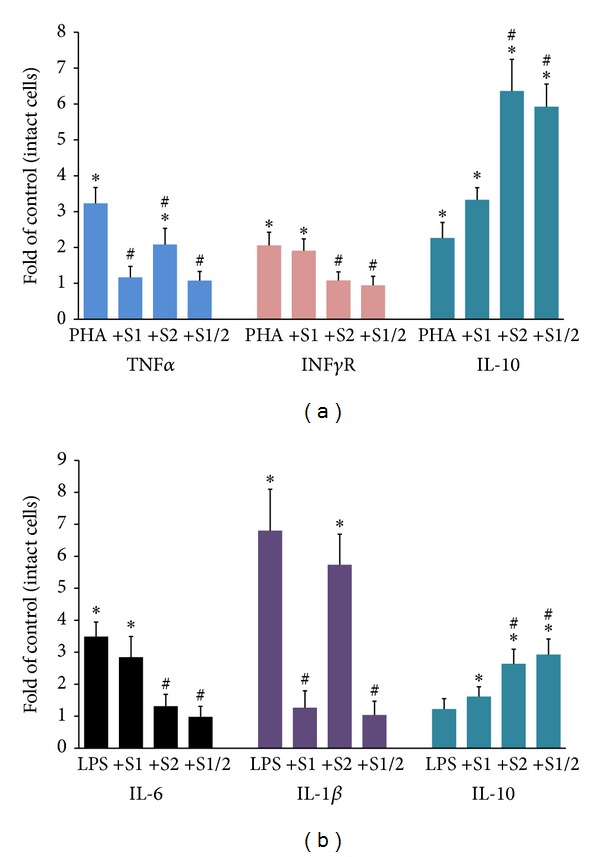
Anti-inflammatory effects of rSLURP-1 and -2 on immunocytes. The anti-inflammatory effects of rSLURP-1 (S1) and -2 (S2), 0.01 *μ*g/mL, on production of proinflammatory cytokines and IL-10 by the CEM stimulated with 10 *μ*M PHA (a) and by the differentiated U937 macrophages stimulated with 200 ng/mL LPS (b) incubated for 16 h in a humid, 5% CO_2_ incubator at a cell density of 1 × 10^6^ cells/well were measured by QIA, as detailed in Materials and Methods. Each experiment was performed in triplicate. Asterisk = *P* < 0.05, compared to intact cells. Pound sign = *P* < 0.05, compared to PHA or LPS given alone.

rSLURP-1 significantly (*P* < 0.05) decreased production of TNF*α* by CEM, downregulated IL-1*β* and IL-6 secretion by U937 cells, and moderately upregulated IL-10 production by both types of immunocytes (Figure 2). rSLURP-2 significantly (*P* < 0.05) downregulated TNF*α* and IFN*γ*R in CEM and reduced IL-6 production by U937 cells (Figure 2). Combining both rSLURPs amplified their anti-inflammatory effects.

## 4. Discussion

Results of the present study demonstrated for the first time that SLURP proteins can produce anti-inflammatory effects by abolishing expression of IBD-related mediators of inflammation in both IEC and immunocytes. These findings suggest that SLURPs may become prototype drugs for the treatment of IBD, because they mimic the inhibitory effect of nicotine and some noncanonical nAChR ligands on gut inflammation. Clinical use of rSLURPs should avoid nicotine-like toxicity, such as off-target and nonreceptor intracellular effects, because SLURPs are the physiological substances produced at low levels by IEC [25] and immunocytes [60] that alter cell functions by acting at nAChRs [46, 47]. Notably, quercetin—a flavonoid that exhibits its nicotinergic activity through *α*3, *α*7, and *α*9 nAChRs [61–64]—produces an anti-inflammatory effect and ameliorates experimental IBD [65, 66].

Both *α*7 and non-*α*7 subtypes of nAChRs might mediate anti-inflammatory effects of rSLURP-1 and -2 in IEC, CEM, and U937 cells. It has been reported that activation of nAChRs inhibits secretion of IL-1*β* and IL-8 in IEC [67, 68]. SLURP inhibition of the production of proinflammatory cytokines in the IEC activated by TLR ligands may have important clinical implication, because compounds inhibiting the immune stimulation involving TLR ligands, especially TLR4, have been reported to be potentially useful for treatment of IBD [31]. Both nicotine and SLURP-1 bind with a high affinity to *α*7 nAChR [46, 69] and both upregulate local production of IL-10 (Figure 2 and [70]), which is otherwise decreased in patients with IBD [71]. T-cells also express *α*4 and *β*2 subunits [20] that could be activated by rSLURP-2. Activation of *α*4*β*2 inhibits immune reactivity [72, 73].

The differences between effects of each rSLURP protein may be due to their predominant action at distinct nAChR subtypes expressed on the cell membrane of different kinds of immunocytes [21, 22] and IEC. By RT-PCR, CCL-241 cells uniquely express *α*3, whereas CCL-248, *α*2 and *α*5, and both cells also express *α*7 and *α*9 nAChRs (data not shown), which is different from the colonic cell line HT29 that carries *α*4-made nAChR [38]. The variations of the nAChR profiles among distinct IEC types help explain regional variations of intestinal responses to smoking/nicotine [4, 70, 74–76].

Previous studies indicated that SLURP-1 can potentiate the ACh action at *α*7 nAChR leading to modifications in functions of cutaneous epithelial cells [77] and immunocytes [78]. Since both IEC and immune cells express this nAChR subtype, the anti-inflammatory effects of SLURP-1 in the gut may result from its action on both cells types simultaneously. Additionally, since SLURP-1 has been shown to upregulate production of ACh by immunocytes [78], this endogenously produced and secreted agonist may further potentiate the *α*7-mediated anti-inflammatory effect of SLURP-1.

## 5. Conclusions

Both rSLURP-1 and -2 inhibit production of inflammatory mediators in human enterocytes, colonocytes, T-cells, and macrophages. Combining both rSLURP proteins amplifies the anti-inflammatory effects. The anti-inflammatory effects of nontoxic nAChR ligands such as SLURPs may therefore ameliorate disease in CD and UC patients. Identification of the predominant types of nAChRs mediating anti-inflammatory effects of each SLURP protein on IEC and immunocytes should help elucidate the intracellular signaling pathways.
